# Altered Lipid Metabolism in Blood Mononuclear Cells of Psoriatic Patients Indicates Differential Changes in Psoriasis Vulgaris and Psoriatic Arthritis

**DOI:** 10.3390/ijms20174249

**Published:** 2019-08-30

**Authors:** Piotr Wójcik, Michał Biernacki, Adam Wroński, Wojciech Łuczaj, Georg Waeg, Neven Žarković, Elżbieta Skrzydlewska

**Affiliations:** 1 Department of Analytical Chemistry, Medical University of Bialystok, 15-089 Białystok, Poland; 2 Dermatological Specialized Center “DERMAL” NZOZ in Bialystok, 15-453 Białystok, Poland; 3 Institute of Molecular Biosciences, University of Graz, 8010 Graz, Austria; 4 LabOS, Rudjer Boskovic Institute, Laboratory for Oxidative Stress, 10000 Zagreb, Croatia

**Keywords:** lipid mediators, psoriasis vulgaris, psoriatic arthritis, lipid peroxidation products, endocannabinoids, eicosanoids

## Abstract

The aim of this study was to investigate possible stress-associated disturbances in lipid metabolism in mononuclear cells, mainly lymphocytes of patients with psoriasis vulgaris (Ps, n = 32) or with psoriatic arthritis (PsA, n = 16) in respect to the healthy volunteers (n = 16). The results showed disturbances in lipid metabolism of psoriatic patients reflected by different phospholipid profiles. The levels of non-enzymatic lipid metabolites associated with oxidative stress 8-isoprostaglandin F2α (8-isoPGF2α) and free 4-hydroxynonenal (4-HNE) were higher in PsA, although levels of 4-HNE-His adducts were higher in Ps. In the case of the enzymatic metabolism of lipids, enhanced levels of endocannabinoids were observed in both forms of psoriasis, while higher expression of their receptors and activities of phospholipases were detected only in Ps. Moreover, cyclooxygenase-1 (COX-1) activity was enhanced only in Ps, but cyclooxygenase-2 (COX-2) was enhanced both in Ps and PsA, generating higher levels of eicosanoids: prostaglandin E1 (PGE1), leukotriene B4 (LTB4), 13-hydroxyoctadecadienoic acid (13HODE), thromboxane B2 (TXB2). Surprisingly, some of major eicosanoids 15-d-PGJ_2_ (15-deoxy-Δ12,14-prostaglandin J_2_), 15-hydroxyeicosatetraenoic acid (15-HETE) were elevated in Ps and reduced in PsA. The results of our study revealed changes in lipid metabolism with enhancement of immune system-modulating mediators in psoriatic mononuclear cells. Evaluating further differential stress responses in Ps and PsA affecting lipid metabolism and immunity might be useful to improve the prevention and therapeutic treatments of psoriasis.

## 1. Introduction

Psoriasis is a chronic autoimmune disease, the most common form of which is psoriasis vulgaris, characterized by pathological interactions between immune cells, especially lymphocytes, and skin cells, especially keratinocytes. In some patients, psoriasis is characterized by a more severe clinical course, leading to the development of psoriatic arthritis [1]. The cause of the disease remains unknown, although some genetic or environmental factors are associated with the development of psoriasis. Thus, it has been observed that lymphocytes and the cytokines they produce, especially interferon γ (IFNγ) and interleukin 17 (IL-17), affect other cells, leading to chronic inflammation and characteristic symptoms such as cutaneous plaques in psoriasis or arthrosis and loss of movement in psoriatic arthritis. Moreover, the pro-inflammatory phenotype of lymphocytes and other immune cells is observed not only locally in the skin or joints, but also in cells isolated from blood, which confirms that general inflammation is present in psoriasis [2]. However, that can only partially explain the course of the disease, still leaving many questions open in respect to the development of psoriasis or the transition of one form to another. Recent studies on immunological diseases have revealed that their development is often accompanied by oxidative stress and increased metabolism of phospholipids, suggesting that both ROS and lipid mediators play important roles in the pathophysiology of psoriasis [3]. In psoriasis, both systemic and local, oxidative stress and increased metabolism of phospholipids have been demonstrated. Among immune-competent, inflammatory cells of psoriatic patients were observed especially for granulocytes, which increasingly generate lipid peroxidation products, thus modulating their inflammatory activities [4]. Furthermore, it has been shown that activation of lymphocytes is also accompanied by the generation of large amounts of reactive oxygen species (ROS) that are important mediators of cytokine-mediated redox biology [5]. Biological activities of ROS are related to their ability to react with biomolecules, such as phospholipids, in particular polyunsaturated fatty acids (PUFA), which leads to lipid peroxidation and generation of reactive aldehydes, e.g., 4-hydroxynonenal (4-HNE), which is denoted also as a “second messenger of free radicals” [6].

In addition to lipid peroxidation, cellular phospholipids are also metabolized by enzymes, the activities of which are increased in various inflammatory processes. The most important enzymes involved in the generation of lipid mediators are phospholipases, cyclooxygenases (COXs), and lipoxygenases (LOXs) [7]. During this process phospholipases begin biosynthesis of endocannabinoids among which the best known are 2-acyloglycerol (2-AG) and anandamide (AEA). Enhanced levels of them are observed in inflammatory diseases like osteoarthritis or systemic lupus erythematosus [8,9]. Endocannabinoids affect cells mostly by interactions with their receptors, cannabinoid receptors (CB1 and CB2). It is known that activation of CB2 receptor results in anti-inflammatory and anti-oxidative regulation, while activation of CB1 shows pro-inflammatory and pro-oxidative activity [10]. In addition, endocannabinoids have been found to be involved in the development of psoriatic comorbidities. Therefore, it can be assumed that disturbances of the endocannabinoid system may also play a significant role in the course of psoriasis.

Moreover, recent studies suggest, that effect of endocannabinoids action is also dependent on the action of their metabolites [11]. Anandamide is metabolized into arachidonic acid which together with arachidonic acid revealed from phospholipids is further metabolized into eicosanoids by (COXs) and LOX. Among cyclooxygenases COX-2 is believed to be the main enzyme responsible for generation of active lipid mediators during inflammation [12], while arachidonic acid is also a substrate to synthesis of thromboxanes, leukotrienes, and prostaglandins [13].

Since lipid mediators are commonly observed in autoimmune diseases, they are constantly gaining increased attention among researchers focused on autoimmune diseases. Lipid mediators might accordingly be important for studies on diseases with poorly understood pathophysiology and without effective therapy, like psoriasis. As such, the aim of this study was to investigate oxidative lipid modifications in mononuclear cells, mainly lymphocytes isolated from patients suffering from psoriasis vulgaris (Ps) or psoriatic arthritis (PsA).

## 2. Results

**Figure 1:**
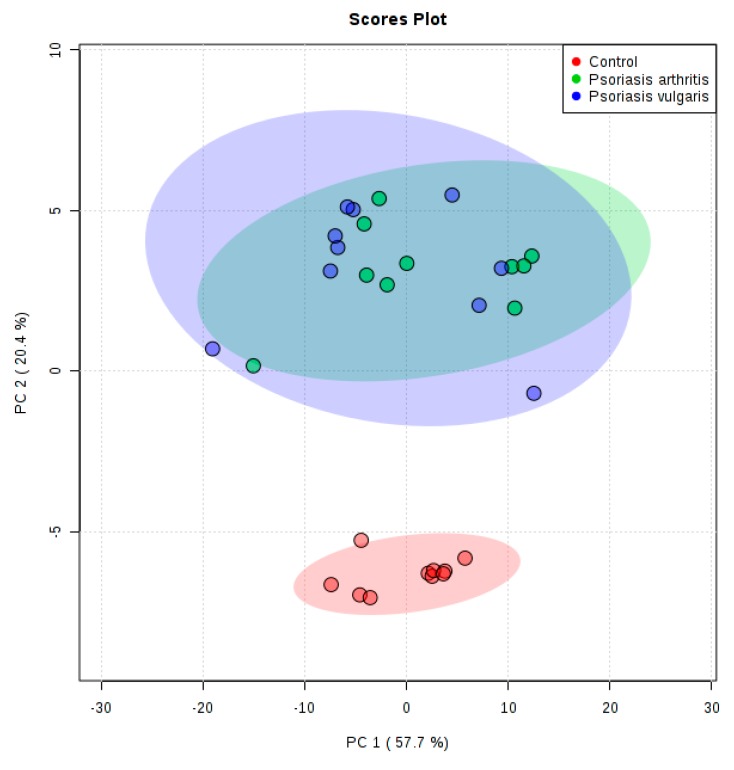
Principal component analysis (PCA) plot of the phospholipid species relative abundance determined by HILIC-LC-MS in lymphocytes of patient with psoriasis vulgaris (n = 10) and psoriatic arthritis (n = 10) as well as healthy subjects (n = 10); 95% confidential intervals are indicated by shaded area.

Phospholipids that could distinguish patients with psoriasis vulgaris and psoriatic arthritis were selected by use of multivariate statistics. Twenty phospholipid species with variable importance in projection (VIP ≥ 1) were selected, which were driving the separation of examined groups. Principal component analysis (PCA) model (Figure 1) was constructed to check classification of each group. The complete data set with the log transformed and auto-scaled phospholipid variables was used to carry out the analysis. The PCA model, on which each point represents an individual sample, showed that the group of healthy subjects was clearly separated from the patients groups, while the separation between both types of psoriasis almost did not occur. The model captured 78.1% of the total variance. The variation in the variables is represented by two principal components of PC1 (57.7%) versus PC2 (14.7%). It is highly probable that PC1 is associated with the age, while PC2 indicates a positive correlation with disease state. It was observed that the samples from both groups of psoriatic patients showed positive values for PC2, whereas those from healthy subjects presented negative values. Finally, we used partial least squares-discriminate analysis (PLS-DA) (Figure S1) and VIP for estimation of the importance of each phospholipid species which drove the separation of the examined groups (Table 1).

Table 1 shows the details of these species that belong to lysophosphatidylcholine (LPC), phosphatidylcholine (PC), phosphatidylinositol (PI), and sphingomyelin (SM) classes. Two phosphatidylcholines containing eicosatrienoic (20:3) and docosadienoic (22:2) acid, namely PC(20:0/22:2), and PC(20:3/24:0), had the highest VIP scores. In general, PC was down-regulated in patients of both types of psoriasis while two SM species, containing tetracosadienoic (24:2) and docosaenoic acid (22:1), respectively, were up-regulated. Other phospholipid species that were changed in the course of both forms of psoriasis belong to the PI class. PI species were down-regulated in both groups of patients with exception of PI(24:0/24:0), PI(24:0/24:1), and PI(18:0/22:0), which were highly up-regulated. Important to notice is that PI species containing arachidonic and linoleic acid, namely PI(16:0/20:4), PI(18:0/20:4), and PI(18:2/22:0) were significantly down-regulated in both groups of psoriatic patients in comparison to healthy subjects. Moreover, most of PI species were found to be more altered in PsA patients than those with Ps. LPC species with the score > 1, namely LPC(16:0) and LPC(18:0), were found to be highly up-regulated in psoriasis in comparison to healthy subjects. Hence, these two LPC species were mostly responsible for the differentiation of both types of psoriasis from healthy subjects (Table 1).

**Figure 2:**
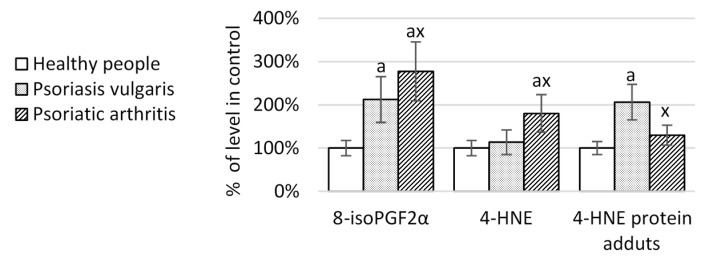
The level of phospholipid oxidative modifications products and of 4-HNE-protein adducts in mononuclear cells of healthy peoples (n = 16) and patients with psoriasis vulgaris (n = 32) and psoriatic arthritis (n = 16). 8-isoPGF2α, F2α-8-isoprostaglandin; 4-HNE, 4-hydroxynonenal. Data points represent the mean ± SD; a, significantly different from healthy subject, *p* < 0.05; x, significantly different from patients with psoriasis vulgaris, *p* < 0.05.

It is often assumed that the reduced level of PUFAs is related to their ROS-dependent and enzyme-dependent metabolism. Consequently, the elevated levels of lipid peroxidation products belonging to compounds generated during PUFAs’ oxidative fragmentation, such as 4-HNE, as well as to compounds generated during PUFAs oxidative cyclisation, such as 8-iso prostaglandin F2α (8-isoPGF2α), was observed, in particular in patients with PsA (Figure 2).

**Figure 3:**
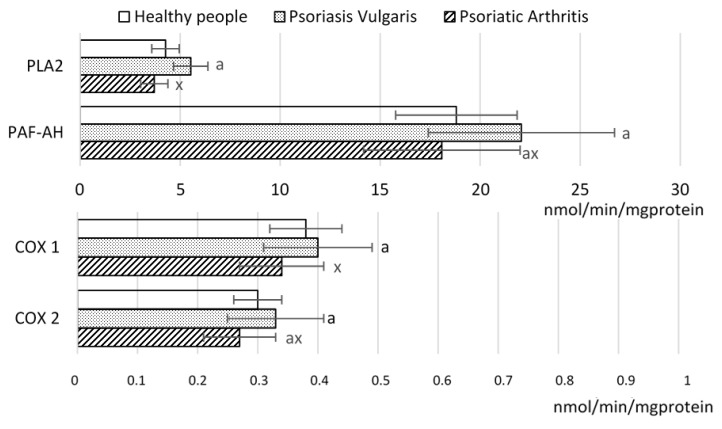
The activity of the enzymes involved in phospholipid metabolism in mononuclear cells of patients psoriasis vulgaris (n = 32) and psoriatic arthritis (n = 16) as well as healthy subjects (n = 16). PLA2, phosholipase A2; PAF-AF, platelet-activating factor acetylhydrolase; COX, cyclooxygenases. Data points represent the mean ± SD; a, significantly different from healthy subject, *p* < 0.05; x, significantly different from patients with psoriasis vulgaris, *p* < 0.05.

That might indeed be that case with psoriatic patients because their observed decrease of PUFAs was associated by enhanced activities of the enzymes metabolizing lipids including phospholipases (PLA2 and PAF-AH), as well as of cyclooxygenase 1 (COX1) and cyclooxygenase2 (COX2), especially in Ps (Figure 3).

It is likely that increased activity of the above listed enzymes promoted generation of endocannabinoids. The levels of anandamide and LEA increased more in mononuclear cells of patients with PsA, while the concentration of 2-AG, 2-LG (2-linoleoylglycerol), and OEA (oleoylethanolamide) was higher in patients with Ps (Table 2). An elevated level of endocannabinoids was observed despite the increased activity of enzymes degrading them (FAAH—fatty acid amide hydrolase and MAGL—monoacylglycerol lipase), especially in mononuclear cells of patients with Ps (Table 2).

**Figure 4:**
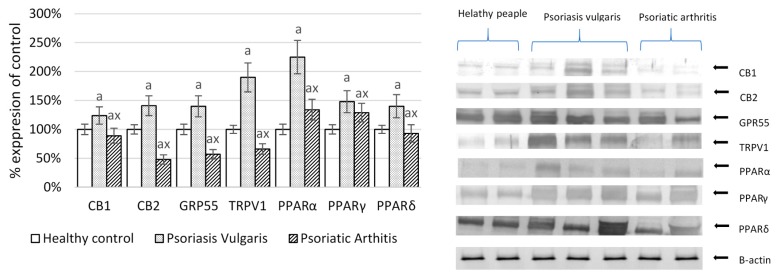
The expression of receptors involved in phospholipid metabolism in mononuclear cells of patients with psoriasis vulgaris (n = 16) and psoriatic arthritis (n = 8) and healthy peoples (n = 8). CB1, CB2, cannabinoid receptors; GRP55, G protein-coupled receptor 55; TRPV, the transient receptor potential cation channel subfamily V member 1; PPAR, peroxisome proliferator-activated receptor. Data points represent the mean ± SD; a, significantly different from healthy subjects, *p* < 0.05; x, significantly different from patients with psoriasis vulgaris, *p* < 0.05.

Increased level of endocannabinoids resulted in enhanced expression of cannabinoids receptors (CB1 and CB2), as well as other receptors activated also by endocannabinoids (GPR55 and TRPV1) in Ps and decreased expression of these receptors in PsA (Figure 4). Products of phospholipid metabolism including endocannabinoids, but also fatty acids and eicosanoids, are ligands of nuclear peroxisome proliferator-activated receptors (PPARα, PPARγ, PPARδ). A significant increase in the expression of these receptors was observed in mononuclear cells obtained from patients with Ps, while it was only moderately raised in mononuclear cells of patients with PsA (Figure 4).

**Figure 5:**
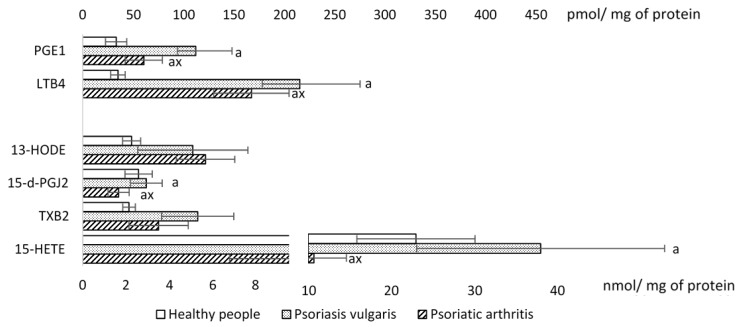
The level of the eicosanoids in mononuclear cells of patients with psoriasis (n = 32), psoriatic arthritis (n = 16), as well as healthy subjects (n = 16). Data points represent the mean ± SD; a, significantly different from healthy subject, *p* < 0.05; x, significantly different from patients with psoriasis vulgaris. Prostaglandin E1 (PGE1), leukotriene B4 (LTB4), 13-hydroxyoctadecadienoic acid (13HODE), thromboxane B2 (TXB2). Surprisingly, some of major eicosanoids 15-deoxy-Δ12,14-prostaglandin J_2_ (15-d-PGJ_2_), 15-hydroxyeicosatetraenoic acid (15-HETE). Data points represent the mean ± SD; a, significantly different from healthy subject, *p* < 0.05; x, significantly different from patients with Ps, *p* < 0.05.

Increased activity of PLA2 lead to the enhanced release of arachidonic acid, which is further metabolized (mainly by cyclooxygenases and lipoxygenases) into another large group of active lipid mediators, notably eicosanoids (Figure 5) Among them, pro-inflammatory factors, such as 13-hydroxyoctadecadienoic acid (13-HODE) and leukotriene B_4_ (LTB4) levels, were increased in both forms of the disease. In particular LTB4 level increased dramatically in psoriasis, especially in Ps. In addition, thromboxane B_2_ (TxB2), (metabolite of pro-inflammatory thromboxane A_2_ (TxA2) level was increased in Ps, which might be related to the activities of cyclooxygenases. The analysis of anti-inflammatory mediators revealed decreased levels of 15-deoxy-Δ12,14-prostaglandin J_2_ (15-d-PGJ_2_) in patients with Ps, while 15-hydroxyeicosatetraenoic acid (15-HETE) levels were slightly, but significantly, increased in Ps, and prostaglandin E1 (PGE_1_) levels were increased both in Ps (four-fold) and in PsA (two-fold).

**Figure 6:**
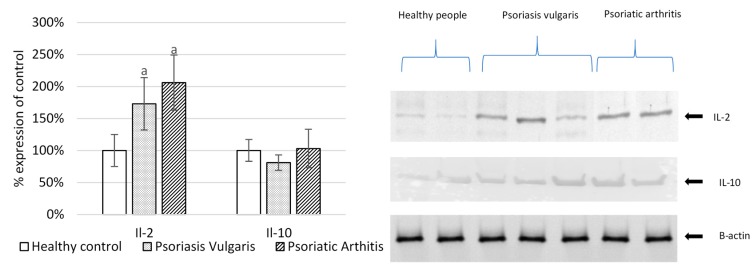
The expression of interleukins and pro-inflammatory mediators in mononuclear cells from patients with psoriasis vulgaris (n = 16), psoriatic arthritis (n = 8), and healthy subjects (n = 8). IL-interleukine. Data points represent the mean ± *SD;* a, significantly different from healthy subjects, *p* < 0.05.

Since psoriasis is known to be associated with inflammation, basic pro-inflammatory and anti-inflammatory parameters were examined (Figure 6). The levels of pro-inflammatory cytokine IL-2 was increased above normal in mononuclear cells of both forms of psoriasis, while anti-inflammatory IL-10 levels were not changed in psoriatic patients.

## 3. Discussion

Psoriasis is a complex inflammatory disease associated with increased activation of lymphocytes [14], which leads to the generation of pro-inflammatory cytokines and to pro-oxidative processes causing oxidative stress [3]. The results of our study confirm the onset of oxidative stress in psoriasis, which is, in particular in PsA, manifested by changes in the metabolism of phospholipids in mononuclear cells, mainly containing lymphocytes, thus offering better understanding of the diseases, due to differentially expressed changes in lipid metabolism between Ps and PsA examined mononuclear cells.

The main source of ROS in cells are usually mitochondria whose respiratory chain may be disrupted by various factors, including phospholipids’ metabolites, such as endocannabinoids, the levels of which were, in the current study, found to be increased in the mononuclear cells of psoriatic patients. It is known that the main endocannabinoids, AEA and 2-AG, may stimulate the function of mitochondria, including the production of hydrogen peroxide by enhanced entry of calcium ions into the cell [15]. In addition, activation of cannabinoid receptors (CB1), caused by endocannabinoids, which was, in our study, also found to be increased in mononuclear cells of psoriatic patients, may promote oxidative stress by enhanced inflammation, e.g., through NOS stimulation leading to increased generation of superoxide and nitric oxide [16]. In favor of this possibility are increased activities of cytosolic NADPH and xanthine oxidases in granulocytes and in plasma of patients with psoriasis, in particular if obtained from patients with PsA [4]. Such changes were accompanied by disturbances in the antioxidant system, which was spread in a stress-response manner lowering the level/activity of cellular antioxidants.

Similarly, redox imbalance and inflammation observed in psoriatic patients were, in the current study, associated with changes in ROS-dependent and enzyme-dependent phospholipid metabolism of mononuclear cells affecting especially PUFAs, which are lipid species the most sensitive to the ROS attack causing lipid peroxidation. Different phospholipid species, including phosphatidylcholines and phosphatidylinositols containing linoleic, arachidonic, eicosatetraenoic and docosadienoic acids, in particular, were down-regulated in mononuclear cells of psoriatic patients. Arachidonic acid and linoleic acid are the major PUFAs, which are oxidized in a ROS-dependent manner to biologically-active lipid mediators, including products of oxidative fragmentation (mainly α,β-unsaturated electrophilic aldehydes, with 4-HNE as the most important one) and products of oxidative cyclisation (mainly prostaglandin derivatives, such as 8-isoPGF2α) that further propagate oxidative damages altering functional activities of the immune-competent cells [17]. The mechanism of 8-isoPGF_2α_ action is based on TNFα activation [18]. So far, increased levels of 8-isoPGF_2α_ have been found in psoriatic patients’ serum [19] as well as in the skin cells [20] being accompanied by elevated TNFα expression [21]. However, 4-HNE is a more potent biomarker of lipid peroxidation, which is involved in complex regulation of inflammatory and immunological responses [6]. The increased levels of the free 4-HNE were observed in our study especially in the mononuclear cells of PsA patients. Opposite to that, the levels of histidine-bound protein adducts of 4-HNE were higher in examined cells of patients with Ps indicating differential metabolic and scavenging pathways for this “second messenger of free radicals” in Ps and in PsA patients. It is known that unsaturated aldehydes, such as 4-HNE, have a strong affinity for covalently linking with nucleophilic amino and thiol groups of biomolecules, including proteins and glutathione (GSH) [17]. Since the thiol groups of protein cysteine residues act as redox switches controlling cell signaling and metabolism [22], versatile interactions of cysteine with signaling molecules, such as 4-HNE, can selectively modulate protein functions [23]. Complementary to that, modifications of protein residues, not only cysteine but also histidine or lysine, may lead to disturbances of biological functions of modified proteins and their translocation to the cell membrane and activation of protein G [24,25,26]. Our findings suggest that such protein adducts may participate in enhanced expression of protein G-coupled receptors (including cannabinoid receptors), as was observed in this study in mononuclear cells of psoriatic patients.

The cellular metabolism of phospholipids, independent of ROS, also includes enzymatic reactions leading to the generation of endocannabinoids dependent on the activity of phospholipases [27]. Despite the increased activity of endocannabinoid-metabolizing enzymes (FAAH and MAGL), endocannabinoid levels were elevated in our patients. The main endocannabinoids, anandamide and 2-arachidonoylglycerol (2-AG), participate in the modulation of inflammation and redox balance mainly through the activation of cannabinoid receptors [28]. Crosstalk between ROS and endocannabinoids has been proven in various diseases [27], but so far not in patients with psoriasis, as was done for the first time in the present study.

In addition, inflammation is associated with increased expression of lymphocyte receptors: GPR55 and TRPV1 [29,30]. According to in vitro studies endocannabinoids themselves may also act as anti-inflammatory agents by promoting the Th2 cell phenotype and further inhibit the production of pro-inflammatory cytokines by lymphocytes [31]. This may suggest that endocannabinoids are generated, among others, to alleviate the inflammatory aspects of psoriasis. In fact, in the case of Ps we observed a higher expression of endocannabinoid receptors but, surprisingly, in the case of PsA, the expression of these receptors has been reduced, suggesting no cellular response to the regulatory role of endocannabinoids. It is possible that the receptors or membrane protein G have been modified by 4-HNE, which can explain also less pronounced increase of the soluble 4-HNE-protein adducts in patients with PsA, who have otherwise higher levels of the free aldehyde than patients with Ps. In any case, the disrupted immunosuppressive effect of endocannabinoids in PsA may be an important factor differentiating the pathophysiology of both forms of psoriasis and may promote exacerbation of Ps into the form of PsA.

Enzymatic degradation of endocannabinoids, with COXs participation in particular, plays a critical role in the onset of the inflammatory cascade with the generation of different biologically-active compounds, including eicosanoids [32]. Among them are various bioactive agents, such as prostaglandins, thromboxanes, and leukotrienes, which can participate in the pathophysiology of psoriasis. Eicosanoids may have opposite inflammatory effects and, thus, modulate skin disorders. The results of this study revealed a significant increase in mononuclear cells of patients with psoriasis TxB2, a stable metabolite of TxA2, which plays a pro-inflammatory role in imiquimod-induced psoriatic dermatitis [33]. Additionally, elevated LTB4 levels may promote skin inflammation [34]. Inflammation may also be modified by 15d-PGJ_2_, which is elevated in Ps, and which inhibits NFκB mediated metabolic pathways by inhibiting IκB kinase (IKK) activity [35].

Another mechanism of interactions redox biology-lipid metabolism in the mononuclear cells of psoriatic patients may be associated with the expression of nuclear receptors like peroxisome proliferator-activated receptors—PPARs [36], whose forms: PPARα, PPARγ, and PPARδ were enhanced in mononuclear cells of patients with psoriasis, particularly with Ps. Moreover, PUFAs, eicosanoids, and endocannabinoids may be ligands of PPARs, which in B and T lymphocytes serve as important regulators of the immune system as potential anti-inflammatory targets for PPAR ligands [37,38].

PPARα and PPARδ show antioxidant properties by suppressing enzymes involved in ROS/RNS generation [39], while enhanced activation of PPARα may also prevent NFκB–dependent inflammation [40]. The results obtained by the murine translation study indicated that PPARα activation may be one of the protective mechanisms against progression of skin inflammation [41]. Additionally, the PPARδ promotes synthesis of antioxidants, such as thioredoxin, superoxide dismutase, or heme oxygenase [42]. Thus, PPARδ enhanced expression seems to be an important antioxidative mechanism in patients with Ps. The significant decrease in their expression observed in PsA confirms that the lack of anti-stress protection against ROS leads to an increase the severity of this disease. In addition, increased PPARγ expression, as discovered in this study, may be involved in reduction of TNFα level, particularly in PsA [43].

## 4. Materials and Methods

### 4.1. Materials

Blood samples were collected from 32 patients with psoriasis vulgaris (Ps) (16 females, 16 men; mean age 38) and 16 patients with psoriatic arthritis (seven females, nine men; mean age 35). Only patients with psoriasis vulgaris for at least six months and characterized by at least 10% of the total body surface affected by the disease were included in the study (the median of their psoriasis surface index and severity index (PASI) was 21, with a range of 15–25). Patients with psoriatic arthritis (PsA) were diagnosed on the basis of a questionnaire CASPAR (ClASsification criteria for Psoriatic Arthritis). All participants gave their informed consent for inclusion in the research. The research was carried out in accordance with the Helsinki Declaration, and the protocol was approved by the Local Bioethics Commission at the Medical University of Białystok (Poland), no. R-I-002/289/2017 (2017.09.28). People receiving topical or oral medications during the four weeks before the study and comorbidities/smoking or alcohol abuse were excluded from examinations.

Blood was collected into tubes with ethylenediaminetetraacetic acid as an anti-coagulant and butylhydroxytoluene as an antioxidant. Density gradient centrifugation (Gradisol L, 300 g, 25 min) was used to obtain mononuclear cells mainly containing lymphocytes fraction from blood. This fraction was washed in phosphate-buffered saline (PBS), then resuspended in PBS containing proteases inhibitors mix. Purity of the cells has been examined microscopically (Nikon Eclipse Ti, Nikon Instruments Inc., Melville, NY, USA). Isolated fractions were lysed by sonification on ice and stored at −80 °C before further analysis. All obtained parameters are expressed per mg of protein (protein levels were examined using Bradford method [44]).

### 4.2. Methods

#### 4.2.1. Phospholipid Profile Estimation

Mononuclear cells from ten patients with Ps, ten patients with PsA (5f + 5m) and ten healthy subjects (5f + 5m) were used to estimate phospholipid profile of each group. Total lipids from all samples were extracted by the modified Bligh and Dyer method [45]. The total amount of phospholipids (PL) was calculated by using a phosphorus assay, performed according to Bartlett and Lewis [46]. Hydrophilic interaction chromatography liquid chromatography-mass spectrometry (HILIC-LC)-MS, performed on an Agilent 1290 ultra-performance liquid chromatography (UPLC) system coupled to an Agilent 6540 quadrupole time of flight mass spectrometer (Agilent Technologies, Palo Alto, CA, USA) was applied to obtain the phospholipid profile. An Ascentis Si HPLC Pore column (15 cm × 1.0 mm, 3 μm; Sigma Aldrich, St. Louis, MO, USA) was used for chromatographic separation in gradient mode. The method was described in details previously [47]. Internal standards PC 14:0/14:0, PI 16:0/16:0, and PE 14:0/14:0 (Avanti Polar Lipids, Alabaster, AL, USA) were used to confirm the ion variations observed in the MS spectra. Identification of each phospholipid species was performed according to the typical fragmentation pathways [48]. The area of each extracted ion chromatogram peak was normalized to the area of an internal standard to calculate relative abundance of each ion. Detailed description of the lipidomic methodology is provided in supplementary materials.

#### 4.2.2. Determination of the Activity of Lipid Metabolizing Enzymes

Spectrophotometric method was used to assay phospholipase A2 (PLA2-EC.3.1.1.4) activity using PLA2 Assay Kit (no. 765021, Cayman Chemical Company, Ann Arbor, MI, USA) according to the kit instructions [49].

PAF acetylhydrolase (PAF-AH-EC.3.1.1.47) activity was measured spectrophotometrically using the Cayman’s PAF Acetylhydrolase Assay Kit (no. 760901, Cayman Chemical Company, Ann Arbor, MI, USA) according to kit instructions [50].

Cyclooxygenase 1 and 2 (COX1/2-EC.1.14.99.1) activities were measured spectrophotometrically using a commercial assay kit (Cayman Chemical Company, Ann Arbor, MI, USA) [51]. For distinguishing COX1 activity from COX2 activity the specific COX1 inhibitor SC-560 was used [52].

#### 4.2.3. 4-HNE Determination

Gas chromatography-mass spectrometry (GC/MS) using the selected ion monitoring (SIM) mode, as the O-PFB-oxime-TMS derivatives, using minor modifications of the method of Luo [53] was used to measure product of phospholipid fragmentation—4-HNE. Transitions of the precursor to the product ion were as follows: m/z 333.0 and 181.0 for 4-HNE-PFB-TMS and m/z 307 for IS (benzaldehyde-D_6+_) derivative. (7890A GC-7000 quadrupole MS/MS, Agilent Technologies, Palo Alto, CA, USA).

#### 4.2.4. 4-HNE-Protein Adducts Determination

The 4-HNE-protein adducts level was measured using ELISA method using anti-4-HNE-His murine monoclonal antibody (genuine anti-4-HNE-Hismurine monoclonal antibody, clone 4-HNE 1g4) and goat anti-mouse antibody (Dako, Carpinteria, CA, USA) as primary and secondary antibodies [54]. Results were shown as a percentage of the expression determined in control cells.

#### 4.2.5. 8-isoPGF2α Determination

LC-MS/MS method of Coolen [55] was used to determination of phospholipid oxidative cyclisation product, 8-isoPGF2α, which was analyzed in negative-ion mode using MRM mode: m/z 353.2→193.1 (for 8-isoPGF2α) and 357.2→197.1 (for 8-isoPGF2α-d4 used as an internal standard) (LCMS 8060, Shimadzu, Kyoto, Japan).

#### 4.2.6. Determination of Endocannabinoids and Enzymes Them Degradation

The LC-MS/MS method was used for the quantification of the endocannabinoids level [56]. Endocannabinoids were analyzed in positive-ion mode using MRM mode. Transitions of the precursors to the product ions were as follows: m/z 348.0→62.15 for AEA, m/z 379.0→269.35 for 2-AG, m/z 356.0→63.05 for AEA-d8, m/z 387.0→294.0 m/z for 2-AG-d8, m/z 430.0→66.0 for OEA-d4, m/z 324.0→62.0 for LEA, m/z 355.0→263.0 for 2-LG, 326.0→62.0 for OEA. (LCMS 8060, Shimadzu, Kyoto, Japan). Results were expressed as amount of cannabinoids per mg of protein. The Siegmund procedure [57] was used to determine FAAH (EC.3.5.1.99) activity, following the releasing of m-nitroaniline (m-NA) from decanoyl m-nitroaniline at 410 nm. Enzyme activity was expressed as the amount of enzyme metabolizing 1 pmol of substrat per minute per mg of protein. Releasing of 5’-thio-2-nitrobenzoic acid from arachidonoyl-1-thio-glycerol was used to spectrophotometric [340 nm] determination of MAGL (EC.3.1.1.23) activity [58]. Enzyme activity was expressed as the amount of enzyme metabolizing 1 pmol of substrate per minute per mg of protein.

#### 4.2.7. Lipid Mediators Determination

Lipid mediators levels: TXB_2_, PGE_1_, 15d-PGJ_2_, 13-HODE, LTB_4_, and 15-HETE were estimated using ultra-performing liquid chromatography tandem mass spectrometry (LCMS 8060, Shimadzu, Kyoto, Japan) [59]. 15-HETE-d_8,_ LTB_4_-d_4_, PGF2α-d_4_ and PGD_2_-d_4_ were used as internal standards for quantification. Lipid mediators were extracted using SPE and analyzed in negative-ion mode (MRM). The precursor to the product ion transition was as follows: m/z 369.3→169.1 for TXB_2_, m/z 353.3→317.2 for PGE_1_, 315.2→271.2 for 15-d-PGJ_2_, m/z 295→277 for 13-HODE, and m/z 319→301.2 for 15-HETE.

#### 4.2.8. Proteins Examination

Western blot analysis of protein expression was performed according to Eissa and Seada [60]. Samples were electrophoretically separated on 10% gels, transferred to 0.2 µm pore-sized nitrocellulose, and incubated overnight with primary antibodies against: GPR55, PPARα (host: rabbit) β-actin (host: mouse) that were purchased from Sigma-Aldrich, (St. Louis, MO, USA). Primary antibodies against: Il-10 (host mouse), CB1, CB2, TRPV1 (host rabbit) were purchased from Santa Cruz Biotechnology (Santa Cruz, CA, USA). Primary antibodies against PPARγ (host: rat) were purchased from Abcam (Cambridge, UK). Primary antibodies against PPARδ were purchased from Invitrogen (Carlsbad, CA, USA). Primary antibodies against IL-2 (host: rat) were purchased from Thermo Fisher Scientific (Thermo Fisher Scientific, Waltham, MA, USA). Next membranes were incubated for 2 h with alkaline phosphatase secondary IgG antibody against corresponding primary antibody (Sigma-Aldrich, St. Louis, MO, USA). Protein bands were visualized using the BCIP/NBT Liquid substrate system (Sigma-Aldrich, St. Louis, MO, USA). To compare the proteins expression between samples, each band intensity was estimated using VersaDoc System and Quantity One software (Bio-Rad Laboratories Inc., Hercules, CA, USA). The results are expressed as a percentage of the expression determined in the control cells.

### 4.3. Statistical analysis

All data were expressed as mean ± SD. For data analysis program Statistica (Statistica 13.3, StatSoft Polska, Cracow, Poland) were used. These data were analyzed using one-way analysis of variance followed by a post hoc Tukey test. Values of *p* < 0.05 were considered significant.

## 5. Conclusions

In conclusion, the results of our research suggest that the development of both forms of psoriasis is strongly associated with oxidative stress resulting in altered lipid metabolism and cytokine production, leading to the development of pro-inflammatory preconditioning of the immune cells. In such a vicious circle, mononuclear cells additionally activate pro-inflammatory states and enhance pro-oxidative, stressful conditions. Thus, ROS and enzyme-dependent changes in mononuclear cell phospholipids can be considered as important processes associated with the pathophysiology of psoriasis. Moreover, the reduced expression of endocannabinoid receptors in PsA suggests the failure of the anti-inflammatory mechanism and may be an important factor leading to the transformation of Ps into a more severe form of PsA. Finally, we assume that monitoring the level of 4-HNE adducts can help predict the progression of Ps into PsA if done complementary to the other parameters of lipid metabolism and the levels of respective cytokines that should be analyzed in further studies.

## Figures and Tables

**Table 1:** Changes in mononuclear cells top 20 phospholipid species with VIP score greater than one comparing the healthy subjects (C; n = 10) to psoriasis vulgaris (n = 10) and psoriasis arthritis (n = 10) groups of patients; ns, not statistically significant.

m/z	RT	ID	Composition	VIP	Log2 (Fold-Change)					
					Control vs. Psoriasis Vulgaris	*p* Value	Control vs. Psoriatic Arthritis	*p* Value	Psoriasis Vulgaris vs. Psoriatic Arthritis	*p* Value
928.6940	18.31	PC(42:2)	PC(20:0/22:2)	1.97	0.63	2.34 × 10^−5^	0.34	1.17 × 10^−3^	0.29	ns
954.7113	18.12	PC(44:3)	PC(20:3/24:0)	1.89	0.54	3.76 × 10^−5^	0.26	2.84 × 10^−3^	0.29	ns
917.6085	3.47	PI(40:2)	PI(18:2/22:0)	1.57	0.68	1.13 × 10^−5^	0.90	1.81 × 10^−8^	−0.21	ns
915.5954	3.47	PI(40:3)	PI(18:0/22:3)	1.57	0.75	1.13 × 10^−5^	0.97	3.91 × 10^−8^	−0.21	ns
1033.7629	3.90	PI(48:0)	PI(24:0/24:0)	1.52	−1.06	5.81 × 10^−4^	−0.85	2.06 × 10^−3^	−0.21	ns
857.6755	18.16	SM(d41:2)	SM(d17:0/24:2)	1.51	−0.50	5.81 × 10^−4^	−0.26	3.36 × 10^−2^	−0.23	ns
868.6052	16.49	PC(38:4)	PC(18:0/20:4)	1.47	−0.181	2.00 × 10^−3^	0.31	1.66 × 10^−2^	0.26	ns
1031.7489	3.90	PI(48:1)	PI(24:0/24:1)	1.44	−1.64	1.03 × 10^−3^	−1.43	1.93 × 10^−3^	−0.2109	ns
857.5159	3.85	PI(36:4)	PI(16:0/20:4)	1.39	0.68	5.81 × 10^−4^	0.89	1.26 × 10^−5^	−0.21	ns
840.5732	16.71	PC(36:4)	PC(16:0/20:4)	1.39	0.56	3.51 × 10^−3^	0.31	3.00 × 10^−2^	0.25	ns
896.6372	16.37	PC(40:4)	PC(18:0/22:4)	1.38	0.42	4.04 × 10^−3^	0.14	ns	0.27	ns
909.5490	3.84	PI(40:6)	PI(18:2/22:4)	1.38	0.61	3.33 × 10^−4^	0.82	2.76 × 10^−6^	−0.21	ns
792.5746	17.39	PC(32:0)	PC(16:0/16:0)	1.37	0.54	4.04 × 10^−3^	0.28	ns	0.25	ns
921.6399	3.99	PI(40:0)	PI(18:0/22:0)	1.36	−1.89	1.63 × 10^−3^	−1.68	3.02 × 10^−3^	−0.21	ns
885.5472	3.83	PI(38:4)	PI(18:0/20:4)	1.35	0.57	2.61 × 10^−4^	−0.26	ns	−0.21	ns
554.3463	20.37	LPC(16:0)		1.33	−1.77	5.81 × 10^−4^	−2.6	3.53 × 10^−6^	0.83	ns
582.3771	20.10	LPC(18:0)		1.31	−1.50	3.32 × 10^−5^	−2.3	7.98 × 10^−8^	0.83	ns
894.6222	16.40	PC(40:5)	PC(18:1/22:4)	1.30	0.50	4.72 × 10^−3^	0.80	ns	0.26	ns
843.6591	18.25	SM(d40:2)	SM(d18:1/22:1)	1.26	−0.50	4.70 × 10^−3^	−0.20	ns	−0.29	ns
911.5644	3.84	PI(40:5)	PI(18:1/20:4)	1.26	0.59	1.63 × 10^−3^	0.24	4.78 × 10^−5^	−0.21	ns

**Table 2:** The level of endocannabinoids and enzymes degrading them (FAAH and MAGL) in mononuclear cells of patients with psoriasis vulgaris (n = 32) and psoriatic arthritis (n = 16) Abbreviations: 2-AG, 2-arachidonoylglycerol; 2-LG, 2-linoleoylglycerol; AEA, anandamide; FAAH, fatty acid amide hydrolase; LEA, dihomo-*γ*-linolenoylethanolamine; MAGL, monoacylglycerol lipase; OEA, oleoylethanolamide. Data points represent the mean ± SD; a, significantly different from healthy subject, *p* < 0.05; x, significantly different from patients with Ps, *p* < 0.05.

Analyzed Parameters	Healthy Subjects	Psoriasis Vulgaris	Psoriatic Arthritis
AEA (pmol/mg protein)	0.17 ± 0.02	0.19 ± 0.03a	0.23 ± 0.04ax
2-AG (pmol/mg protein)	1.87 ± 0.18	3.24 ± 0.45a	2.78 ± 0.37ax
2-LG (pmol/mg protein)	5.59 ± 0.87	7.92 ± 0.99a	7.41 ± 0.93a
LEA (pmol/mg protein)	0.79 ± 0.08	0.94 ± 0.15a	1.17 ± 0.15ax
OEA (pmol/mg protein)	0.33 ± 0.05	0.47 ± 0.07a	0.36 ± 0.05a
FAAH (pmol/min/mg protein)	170 ± 16	219 ± 28a	242 ± 31ax
MAGL (pmol/min/mg protein)	55 ± 6	75 ± 10a	83 ± 14ax

## Supplementary Materials

Supplementary materials can be found at https://www.mdpi.com/1422-0067/20/17/4249/s1

## Abbreviations

2-AG	2-Arachidonylglycerol
2-LG	2-Linoleoylglycerol
4-HNE	4-Hydroxynonenal
4-ONE	4-Oxynonenal
8-isoPGF2α	8-iso prostaglandin F2α
13-HODE	13-hydroxyoctadecadienoic acid
15-HETE	15-Hydroxyeicosatetraenoic acid
15d-PGJ_2_	15-deoxy-Δ12,14-prostaglandin J_2_
AEA	Anandamide
AIDS	Acquired immunodeficiency syndrome
CB	Cannabinoid receptor
COX	Cyclooxygenase
EDTA	Ethylenediaminetetraacetic acid
ELISA	Enzyme-linked immunosorbent assay
ERK5	Extracellular-signal-regulated kinase 5
FAAH	Fatty acid amide hydrolase
GC	Gas chromatography
GPR55	G protein-coupled receptor 55
HILIC	Hydrophilic interaction chromatography
HPLC	High-performance liquid chromatography
IFNγ	Interferon γ
IKK	IκB kinase
IL	Interleukin
IMQ	Imiquimod
LC	Liquid chromatography
LEA	Dihomo-γ-linolenoylethanolamine
LOX	Lipoxygenases
LPC	Lysophosphatidylcholine
LTB_4_	Leukotriene B4
MAGL	Monoacylglycerol lipase
MS	Mass spectrometry
NFκB	Nuclear factor kappa-light-chain-enhancer of activated B cells
NOX	NADPH oxidase
Ns	Not significant
OEA	Oleoylethanolamide
PAF-AH	Platelet-activating factor acetylhydrolase
PBS	Phosphate-buffered saline
PC	Phosphatidylcholine
PCA	Principal component analysis
PGE1	Prostaglandin E1
PI	Phosphatidylinositol
PLA2	Phospholipase A2
PLS-DA	partial least squares-discriminate analysis
PPAR	Peroxisome proliferator-activated receptor
Ps	Psoriasis vulgaris
PsA	Psoriatic arthritis
PUFAs	Polyunsaturated fatty acids
ROS	Reactive oxygen species
RT	Retention time
SIM	Selected ion monitoring
SLE	Systemic lupus erythematosus
SM	Sphingomyelin
Th	T helper lymphocytes
TNFα	Tumor necrosis factor α
TNFR	Tumor necrosis factor receptor
TRPV1	The transient receptor potential cation channel subfamily V member 1
TxA2	Thromboxane A2
TxB2	Thromboxane B2
UPLC	Ultra-performance liquid chromatography
VIP	variable importance in projection
