# Supplementary material for: Altered Lipid Metabolism in Blood Mononuclear Cells of Psoriatic Patients Indicates Differential Changes in Psoriasis Vulgaris and Psoriatic Arthritis
Source: Int J Mol Sci. 2019 Aug 30;20(17):4249. doi: 10.3390/ijms20174249 (PMC6747546; doi:10.3390/ijms20174249)
Supplement: Supplementary file 1 [file ijms-20-04249-s001.pdf]

SUPPLEMENTARY MATERIALS:

Methods

Phospholipid profile-lipidomic examination.

Lipid Extraction

Lipids were extracted from all samples using the modified Bligh and Dyer method [16]. In brief, a pellet of the cells (~107 cells) obtained after centrifugation at 8000 rpm for 5 min was re-suspend in 1 mL of milliQ water. Then, 3.75 mL of a 1:2 (*v*/*v*) CHCl3: MeOH solution was added to each samples and vortexed and incubated on ice for 60 min. After the incubation period, 1.25 mL of chloroform was added, vortexed, and 1.25 mL of H_2O (milliQ) was added to obtain a two-phase system. Finally, lipid extracts were recovered from the organic phase after centrifugation at 2500× *g* for 10 min at room temperature, evaporated to dryness with a nitrogen stream, and stored at –20 °C before analysis.

Characterization of the Phospholipid Profile by LC-MS.

Hydrophilic interaction liquid chromatography (HILIC-LC)-MS, performed on an UPLC system (Agilent 1290, Agilent Technologies, Santa Clara, CA, USA) coupled to a quadrupole time-of-flight mass spectrometer (Agilent, QTOF 6540) was used for PL classes separation. Mobile phase A consisted of 25% water, 50% acetonitrile, and 25% (*v*/*v*) methanol, with 10 mM ammonium acetate. Mobile phase B consisted of 60% acetonitrile and 40% methanol with 10 mM ammonium acetate. Total lipid extracts were diluted in mobile phase A, and 5 μl of the reaction mixture was introduced into an Ascentis Si HPLC Pore column (15 cm × 1.0 mm, 3 μm; Sigma–Aldrich). The solvent gradient was programmed as follows: the gradient started with 0% of A and linearly increased to 100% of A for 20 min, and was held isocratically for 35 min, returning to the initial conditions in 5 min. The flow rate through the column was 30 μl/min. Phospholipid molecular species were identified according to their typical fragmentation pathways [18]. Internal standards PC 14:0/14:0, PI 16:0/16:0, and PE 14:0/14:0 (Avanti Polar Lipids) were used to confirm the ion variations observed in the MS spectra according to lipid maps [1]. The relative abundance of each ion was calculated by normalizing the area of each extracted ion chromatogram peak to the area of an internal standard. Relative abundances of each ion were calculated by normalizing the area of each peak to the peak area of an internal standard.

Electrospray Mass Spectrometry Conditions

The characterization of PL classes and individual phospholipid molecular species within each class was achieved by data-dependent ESI–QTOF–MS and MS/MS in negative mode (PI, PE) with the formation of $[M\text{-}H]^-$ and (PC, LPC, SM) with the formation of $[M\text{+}OAc]^-$ in an electrospray (ESI) quadrupole time-of-flight (Agilent, QTOF 6540) mass spectrometer (Agilent, Santa Clara, CA, USA). ESI (Agilent Dual AJS ESI) conditions in electrospray mass spectrometry QTOF (Agilent) were as follows: the electrospray voltage was 3.0 kV in negative mode, the capillary temperature was 250 °C, and the sheath gas flow was 13 L/min. Parent scan spectra were acquired between m/z 100 and 1500. Collision energy was fixed at 35 for MS/MS. Data acquisition was carried out with Mass Hunter data software (version B0.8.0). An isolation width of 1.3 Da was used for the MS/MS experiments.

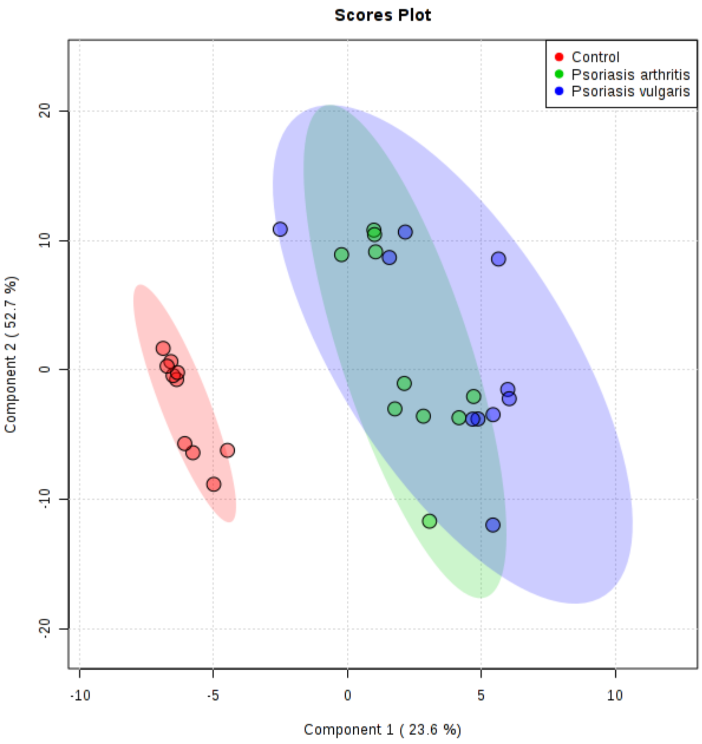

Figure S1. Partial least squares-discriminate analysis (PLS-DA) plot of the phospholipid species relative abundance determined by HILIC-LC-MS in lymphocytes of healthy people (n = 10) and both groups of psoriatic patients (n = 10; n = 10); 95% confidential intervals are indicated by the shaded area.

References

1. Ivanova, P.T.; Milne, S.B.; Byrne, M.O.; Xiang, Y.; Brown, H.A. Glycerophospholipid identification and quantitation by electrospray ionization mass spectrometry. *Meth. Enzym.* **2007**, *432*, 21–57.
16. Carney, S.T.; Lloyd, M.L.; MacKinnon, S.E.; Newton, D.C.; Jones, J.D.; Howlett, A.C.; Norford, D.C. Cannabinoid Regulation of Nitric Oxide Synthase I (nNOS) in Neuronal Cells. *J. Neuroimmune Pharm.* **2009**, *4*, 338–349.
18. Timmermann, M.; Högger, P. Oxidative stress and 8-iso-prostaglandin F(2alpha) induce ectodomain shedding of CD163 and release of tumor necrosis factor-alpha from human monocytes. *Free Radic. Biol. Med.* **2005**, *39*, 98–107.
